# Genetic Architecture of Contemporary Adaptation to Biotic Invasions: Quantitative Trait Locus Mapping of Beak Reduction in Soapberry Bugs

**DOI:** 10.1534/g3.113.008334

**Published:** 2013-12-17

**Authors:** Y. Yu, Jose A. Andrés

**Affiliations:** Department of Biology, University of Saskatchewan, Saskatoon, SK S7N-5E2, Canada

**Keywords:** quantitative genetics, rapid evolution, diversification, host association, hemiptera

## Abstract

Biological invasions can result in new selection pressures driven by the establishment of new biotic interactions. The response of exotic and native species to selection depends critically on the genetic architecture of ecologically relevant traits. In the Florida peninsula, the soapberry bug (*Jadera haematoloma*) has colonized the recently introduced Chinese flametree, *Koelreuteria elegans*, as a host plant. Driven by feeding efficiency, the populations associated with this new host have differentiated into a new bug ecomorph characterized by short beaks more appropriate for feeding on the flattened pods of the Chinese flametree. In this study, we have generated a three-generation pedigree from crossing the long-beaked and short-beaked ecomorphs to construct a *de novo* linkage map and to locate putative quantitative trait locus (QTL) controlling beak length and body size in *J. haematoloma*. Using amplified fragment-length polymorphism markers and a two-way pseudo-testcross design, we have produced two parental maps in six linkage groups, covering the known number of chromosomes. QTL analysis revealed one significant QTL for beak length on a maternal linkage group and the corresponding paternal linkage group. Three QTL were found for body size. Through single marker regression analysis, nine single markers that could not be placed on the map were also found to be significantly associated with one or both of the two traits. Interestingly, the most significant body size QTL co-localized with the beak length QTL, suggesting linkage disequilibrium or pleiotropic effects of related traits. Our results suggest an oligogenic control of beak length.

Adaptive evolution plays a key role in biotic invasions. When a species is introduced to a new habitat, it is likely to experience new selective pressures, and populations of invaders frequently experience rapid evolutionary changes (*e.g.*, Mooney and Cleland 2001; Lee 2002; Lambrinos 2004; Suarez and Tsutsui 2008; Shine 2012). Simultaneously, invaders also act as selective agents, often driving evolutionary changes in the exposed native populations (reviewed in Strauss *et al.* 2006). Both evolvability (*i.e.*, the ability of the genetic system to produce and maintain potentially adaptive genetic variants; Hansen 2006) and the response of invasive and native species to selection depend critically on the genetic architecture of ecologically relevant traits (see Colautti *et al.* 2010, 2012).

There are three main approaches to measuring the genetic architecture of functional traits. One measure is the G matrix (Lynch and Walsh 1998), which is composed of genetic variances and covariances among traits sharing developmental and genetic processes. The G matrix can rapidly evolve in natural populations (Doroszuk *et al.* 2008). However, the only study comparing G matrices between native and invasive populations (Calsbeek *et al.* 2011) to our knowledge found similar molecular-genetic underpinnings of the matrix elements between invasive and native populations. A second measure of genetic architecture is the estimate of the relative effects of additive and nonadditive (dominant, epistatic, and pleiotropic) genetic variances on individual traits. Although generally it is assumed that the response to selection relies only on the existence of additive genetic variance, gene interactions may play a central role in contemporary evolution because directional epistasis makes gene effects become evolvable and enables rapid changes in additive effects and evolvability (Carter *et al.* 2005; Hansen 2006). In invasive species, nonadditive genetic variance seems to play a key role during the colonization of new habitats (see Lee 2002). Similarly, research on native phytophagous insects shifting onto introduced hosts has highlighted the role of epistasis and other non-additive genetic effects in the rapid colonization of the invasive hosts (Carroll *et al.* 2001, 2003; Carroll and Loye 2012). The third measure of genetic architecture is the dissection of trait variation into its genomic components facilitated by advances in molecular genetics. Quantitative trait locus (QTL) mapping can reveal the number and type of genomic regions, and potentially genes, affecting quantitative variation as well as the number of possible gene interactions. To date, only a few studies have used QTL to look at the genetic basis of “invasiveness” (Linde *et al.* 2001; Weinig *et al.* 2007) and to our knowledge QTL mapping has not yet been used to look at evolutionary responses of native species to invasions.

Host shifts of phytophagous insects represent the best body of evidence for the rapid evolution of native species in response to the introduction of novel species (Strauss *et al.* 2006). In this article, we focus on an anthropogenic host-shift in the soapberry bug, *Jadera haematoloma*, and use a QTL approach to study the genetic architecture of “beak” length, a complex, heritable trait that is closely associated with the species’ ability to colonize new hosts.

## Adaptations to biotic invasions in soapberry bugs

Soapberry bugs comprise a subfamily of three widespread genera of seed predator bugs that have become a textbook example of evolution in action (*e.g.*, Moore and Moore 2006; Futuyma 2013; Freeman and Herron 2013). These insects exploit a broad variety of host plants from the family Sapindaceae (Carroll 2007). In North America and Australia, different species of soapberry bug show ongoing rapid evolution of their mouthparts (stylets or “beaks”) to better match the seed defense structures of newly introduced hosts (Carroll and Boyd 1992; Carroll *et al.* 1997; Dingle *et al.* 2009). On the Florida peninsula, populations of the Neotropical soapberry bug *J. haematoloma* feed on the seeds of both the native balloon vine (*Cardiospermum corindum*) and the invasive Chinese flametree (*Koelreuteria elegans*), which was introduced into urban areas about 70 years ago. These two hosts differ in fruit size, phenology, and seed chemical defenses (Seigler and Kawahara 1976; Carroll and Boyd 1992; Carroll *et al.* 1998, 2003). Driven by selection as the result of these differences the populations feeding on the newly colonized tree (*K. elegans*) have evolved into the “derived” ecomorph. Several morphological, physiological, and behavioral differences exist between the ancestral and derived *J. haematoloma* ecomorphs. Possibly the most striking one is the reduction of beak length appropriate to exploit the flatter fruits of the invasive tree (Carroll *et al.* 1998, 2001, 2003; Dingle *et al.* 2009). Controlled crosses, common garden and artificial selection experiments have shown that beak size differences are heritable, that beak length is controlled by multiple genes, and that epistatic interactions are likely to play a key role in the evolution of shorter beaks (Carroll *et al.* 2001; Carroll 2007; Dingle *et al.* 2009). This study represents the first attempt to identify the location, number, and effect of the genomic regions associated with beak length, a trait that plays a central role in the trophic diversification of heteropterans.

## Materials and Methods

### Mapping population

**Figure 1:**
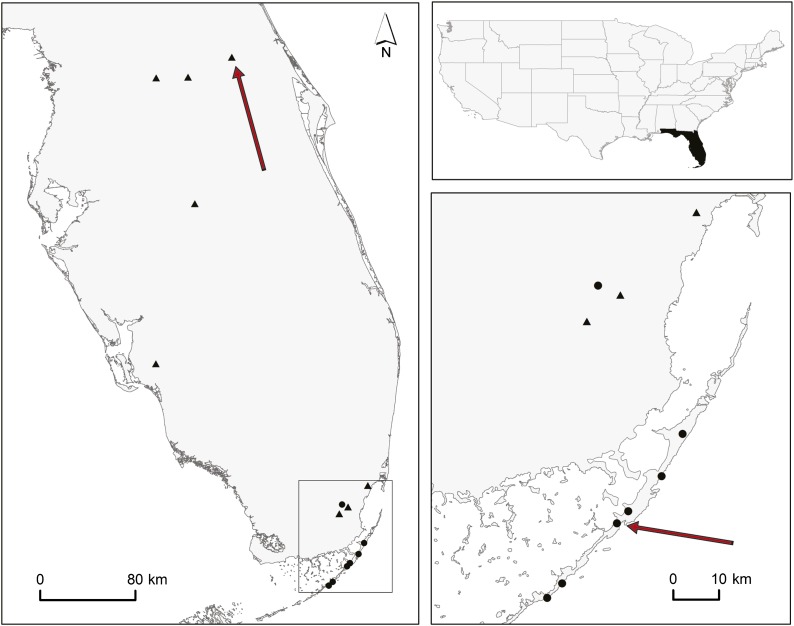
*Jadera haematoloma* populations and sampling sites. Upper right: map of the United States of America with Florida highlighted. Left: Florida. Lower right: close-up of the Florida Keys and part of the tip of the peninsula. Dots represent known populations of the ancestral long-beaked ecomorph feeding on the native balloon vine (*C. corindum*). Triangles represent known populations of the derived short-beaked ecomorph feeding on the introduced Chinese flametree (*K. elegans*). Arrows indicate the sampling sites where the founding pair of the experimental cross were sampled.

For this study, we collected soapberry bugs in two allopatric populations in Florida (Figure 1). In Key Largo (25° 6′ 11.40′′, −80° 26′ 2.88′′), we collected individuals with long beaks feeding on the native balloon vine (*Cardiospermum corindum*). We collected short beak individuals feeding on the introduced Chinese flametree (*Koelreuteria elegans*) in a locality near Orange City (northern Florida; 28° 57′’ 8.52′′ −81° 18′ 19.50′′). The Euclidean distance between these two populations is 437 km. Therefore, although adult bugs are relatively good flyers, gene flow between these two populations is likely to be negligible.

Field-collected individuals were maintained in the laboratory on commercially available seeds of *Koelreuteria paniculata* under controlled light and temperature conditions similar to those of the field collection sites (13.5 hr of daylight at 29°, 10.5 hr of night time at 20°, fluorescent tubes). Previous second generation cross-rearing experiments have shown that rearing long-beaked (ancestral ecomorph) individuals on Koelreuteria seeds affects their developmental time but has little effects on the beak length of females (Carroll *et al.* 1997, 2001). For mapping purposes we produced an F2 mapping population from a single pair of F1 full sibling in a cross between a first-generation, lab-reared, long-beaked female and short-beaked male. We sexed the resulting offspring at adulthood and measured the body and beak (labial) lengths using a digitally calibrated Leica MZ16A stereomicroscope (retest correlation = 0.998). All individuals were then stored at −20° in 100% ethanol for subsequent genetic analyses.

### Fluorescent amplified fragment-length polymorphism (AFLP) methods

We developed AFLP markers following Vos *et al.* (1995) with fluorescently labeled primers (Hartl and Seefelder 1998; Huang and Sun 1999; Ashikawa *et al.* 1999; Trybush *et al.* 2006). For each individual bug, we extracted genomic DNA from the thorax using MasterPure DNA Purification Kit (Epicentre) following manufacturer’s instructions. Approximately 100 ng of DNA of each sample was digested with 5 U *Eco*RI and 5 U *Mse*I (New England BioLabs) for 30 min at 37° in 1x NEB buffer 4 and bovine serum albumin in a total reaction volume of 30 μL. Next, to ligate the resulting fragments to the adapters, we added 0.5 µM *Eco*RI adapter, 5 µM *Mse*I adapter, and 60 cohesive end units of T4 DNA ligase (New England Biolabs) in a total volume of 10 µL to the 30 μL of digestion reaction mixture. After incubation (30° for 90 min), we diluted the samples 10 times with double-distilled water and used 2.5 µL of each sample as a template to conduct the preselective polymerase chain reactions (PCRs) in a total reaction volume of 10 µL (1x PCR buffer, 0.5 μM each of either *Eco*RI-C or *Eco*RI-G combined with each of *Mse*I-C, *Mse*I-G, or *Mse*I-TC primers, 0.2 mM dNTPs, and 0.5 U QIAGEN Top-Taq DNA polymerase; 72° for 150 sec followed by 94° for 3 min, then 22 cycles of [94° for 30 sec, 56° for 1 min, and 72° for 1 min], and finally 72° for 10 min). We diluted these preamplified products 1:20 and used them as template for selective PCR amplifications in 10 µL (1x PCR buffer, 0.5 μM of an *Eco*RI selective primer and an *Mse*I selective primer, 0.2 mM dNTPs, and 0.5 U QIAGEN Top-Taq DNA polymerase) using a touchdown protocol (95° for 3 min, 13 cycles of [94° for 30 sec, 65° for 30 s with −0.7°/cycle, and 72° for 1 min], 12 cycles of [94° for 30 sec, 56° for 30 sec, and 72° for 1 min], and finally 72° for 10 min). After prescreening, we selected 16 combinations of *Eco*RI ^6-FAM^−*Mse*I primers that generated clear fluorescence peaks (CTC-CAAG, CTC-CCTA, CTC-CGAC, CTC-CTGC, GAC-CAAG, GAC-CCTA, GAC-CGAC, GAC-CTGC, CAT-GGAT, CAT-GATC, CAT-GCCA, CAT-GTTC, CCA-TCCA, CCA-TCGC, CCA-TCAT, CCA-TCTG). These primers are similar to those designed for other insect species but *Mse*I primers contained four selective nucleotides to help reduce fragment size homoplasy. To assess the reproducibility of our fingerprinting method, the aforementioned protocol (including DNA extractions) was repeated on both parents and grandparents. Only clearly repeatable peaks were used in the construction of the map.

To prepare DNA fragments for separation by capillary electrophoresis, a sample loading solution was prepared by mixing 0.1 μL of 600-LIZ size standard (Applied Biosystems) with 8.9 μL of Hi-Di Formamide (Applied Biosystems), and 1 μL of 1:30 dilution of selective PCR amplification product. Samples were analyzed in ABI 3130*xl* Genetic Analyzer (Applied Biosystems). The presence or absence of fragments was initially scored automatically using GeneMapper v4.1 (Applied Biosystems) with a minimum relative fluorescence unit of 30; other parameters were left at default. To further reduce size homoplasy we only scored fragments within the 90–550 bp size range (Caballero *et al.* 2008; Paris *et al.* 2010). Bin and peak calls were then confirmed upon manual inspection.

### Genetic linkage analysis and map construction

Polymorphic, repeatable AFLP markers were classified into different segregation classes depending on the allele patterns of the parents. In total, we defined three marker classes using the CP (outbreeding species full-sibling family) population type implemented in JoinMap 4.0 (Van Ooijen 2006): (1) markers that segregate only in the mother (*lm × ll*), (2) markers that segregate only in the father (*nn × np*), and (3) markers that segregate in both parents (*hk × hk*). The expected segregation ratios were 1:1 for the first two classes and 3:1 for the last one. To evaluate any discrepancy from the expected segregation ratios we used the χ^2^ goodness-of-fit method as implemented in JoinMap 4.0. Markers showing segregation distortion at the significance level of *P* = 0.05 were excluded from further analyses. Linkage groups were determined using a logarithm of odds (LOD) threshold of 4.0. Map construction was performed using the Kosambi mapping function and the regression mapping algorithm. Two independent (maternal and paternal) maps were generated using *lm × ll* and *nn × np* markers, respectively, employing a two-way pseudo-testcross strategy (Grattapaglia and Sederoff 1994). The positions of these markers were taken to be fixed orders to further populate the parental maps with *hk* × *hk* markers segregating in both parents. The *hk* × *hk* markers were then used to compare maternal and paternal linkage groups. To test whether the AFLP markers were randomly distributed within linkage groups we used the χ^2^ goodness-of-fit method proposed by Rouppe van der Voort *et al.* (1997).

### QTL analysis

For our QTL analyses, we used the BCF2 module of GridQTL (Seaton *et al.* 2006) available online at http://www.gridqtl.org.uk. The statistical approach of this module adopts the methods of Haley *et al.* (1994). It is suitable for crosses between outbred lines and assumes that the alternative alleles at major QTL affecting the traits of interest are fixed (*e.g.*, lineages with different selection histories). QTL analyses using the TREE module, which does not assume fixed QTL, found QTL of similar size on the same linkage groups (data not shown). Significance thresholds were obtained from permutation tests (n = 10,000) as described in Churchill and Doerge (1994). We considered a QTL significant if it was detected at either *P* < 0.01 at the chromosome-wide level or *P* < 0.05 at the experiment-wide level. We considered a QTL suggestive if it was only detected at *P* < 0.05 at the chromosome-wide level.

We used a forward and backward selection interval mapping approach for QTL analysis (Guo *et al.* 2008; Leach *et al.* 2012): First, a one-QTL model that included the additive and dominant effects of a QTL was fitted at each 1 cM by least square methods for beak and body lengths. If one or more significant or suggestive QTL were detected, the one showing the highest *F*-value was considered to be the first QTL. Second, by using the first QTL as genetic background effects, we searched for QTL of lesser effect in the other linkage groups. In addition, a two-QTL model was fitted to detect any other potential QTL on the same linkage group. Among the significant or suggestive QTL detected at this step, the one with the greatest *F*-value was considered as the second QTL. Next, in the backward selection step, we used this new QTL as genetic background effects to re-estimate the position and effects of the first one. Adjusted parameters of the first QTL were used as genetic background effects and the second QTL was again reassessed. The forward and backward steps were iterated until the parameters for the two identified QTL remained constant. Third, the parameters of the two QTL were used to detect any new QTL. The previous steps were repeated until no new significant or suggestive QTL were found when using all previously detected QTL as genetic background. Finally, we estimated the phenotypic variance explained by each QTL according to the equation of Wang *et al.* (2012).

In addition, we also conducted single-marker regression analysis on markers that were excluded due to segregation distortion, and markers that failed to be grouped with the current linkage groups at an LOD threshold of 4.0 (unlinked). For each marker, phenotypic values (beak or body length) were separated into two groups based on the genotypes (presence or absence of the AFLP fluorescence peak), and analysis of covariance, taking sex as a covariate, was used to find significant difference at *P* < 0.05. For those markers found to be significant, we estimated the percentage of phenotypic variance explained by each marker using the equation:

$${\text{V}}_{\text{EXPLAINED}}={\text{SS}}_{\text{marker}}/\left({\text{SS}}_{\text{total}}-{\text{SS}}_{\text{sex}}\right)\times 100\%$$

Where SS_marker_ is the sum of squares absorbed by the marker after adjusting for the covariate sex in the full model, SS_total_ is the corrected total sum of squares in the null model, and SS_sex_ is the sum of squares absorbed by sex alone in the reduced model.

To detect any potential QTL × sex interactions, we included a sex interaction term into the model and we estimated both additive and dominance effects of the QTL in each sex using GridQTL. We considered that significant sex differences in the estimates of the QTL effects are indicative of QTL × sex interactions. Finally, to detect QTL with epistatic effects, we first imported the genotypic probabilities for each 1 cM calculated by GridQTL into R/qtl using outbred.qtl (R package; Nelson *et al.* 2011). Then we examined genome-wide evidence for epistasis using the *scantwo* function of R/qtl with the Haley-Knott regression method. LOD significance thresholds were determined by permutation tests (n = 500).

## Results and Discussion

### Segregation patterns

**Figure 2:**
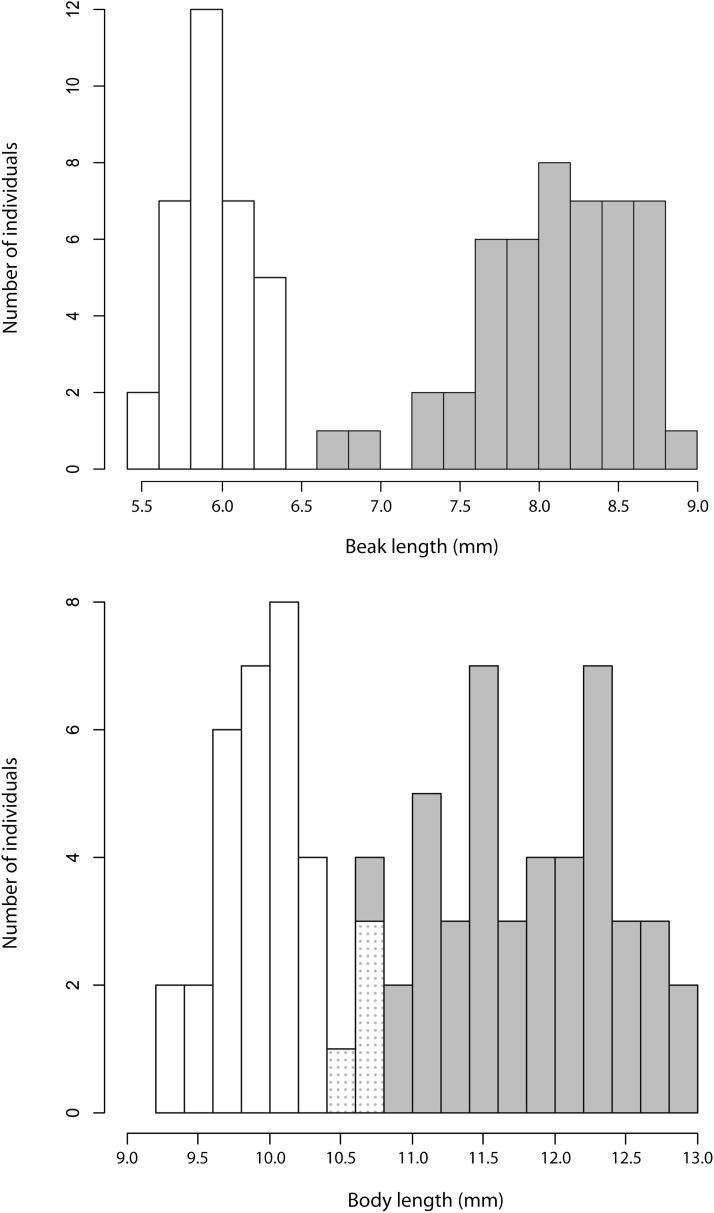
Histograms showing beak and body distributions in the F2 generation. Shaded bars indicate females; white bars indicate males. The dotted areas indicate the overlap between the two.

To generate a linkage map, we produced 81 F2 individuals (48 females and 33 males) from a single F1 cross (female: beak length = 7.57 mm, body size = 12.37 mm; male: beak length = 5.83 mm, body size = 8.57 mm) between two parental diverging lineages of *J. haematoloma* associated with two different host plants (long-beaked female: beak length = 8.11 mm, body size = 12.38 mm; short-beaked male: beak length = 5.82 mm, body size = 10.12 mm). This species is sexually dimorphic (Carroll and Boyd 1992). Accordingly, the resulting female offspring were on average bigger (mean ± SD: 11.75 mm ± 0.64 mm) than the male offspring (9.99 mm ± 0.36 mm), and female beaks (8.09 mm ± 0.48 mm) were on average longer than those of the males (5.93 mm ± 0.23 mm; Figure 2 and Supporting Information, File S2). As expected, the observed distribution of beak sizes in the experimental cross is intermediate between those observed in natural populations of the parental lineages (see Carroll and Boyd 1992).

The 16 primer combinations used in this study resulted in more than 1400 AFLP fragments between 90 and 550 bp. Only polymorphic fragments that could be scored unambiguously (n = 287) were pre-selected for the construction of the linkage map (see File S1). However, a total of 73 of these preselected markers showed significant segregation distortion from the 1:1 or 3:1 expected ratios (χ^2^ test, *P* < 0.05) and were excluded from the linkage analysis. Of the remaining markers, a total of 65 (30.4%) markers were heterozygous in the F1 female (coded as *lm* × *ll*), 47 (22.0%) in the F1 male (coded as *nn* × *np*), and 102 (47.7%) were heterozygous in both parents (coded as *hk* × *hk*).

**Figure 3:**
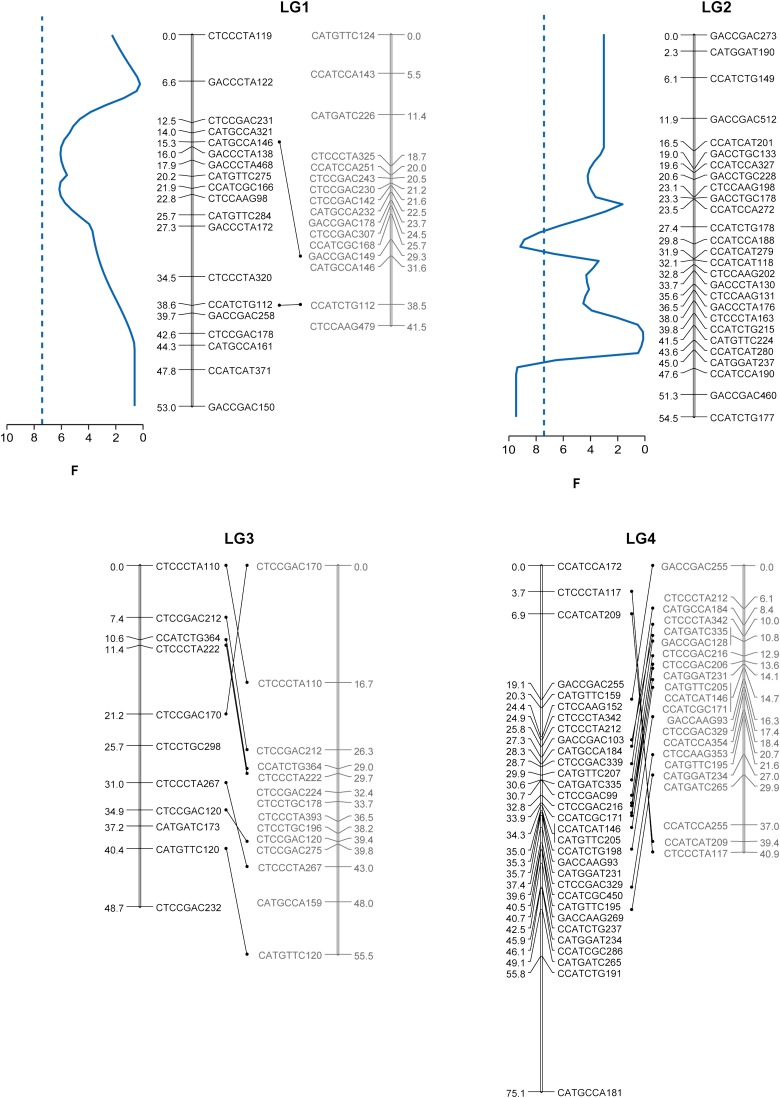

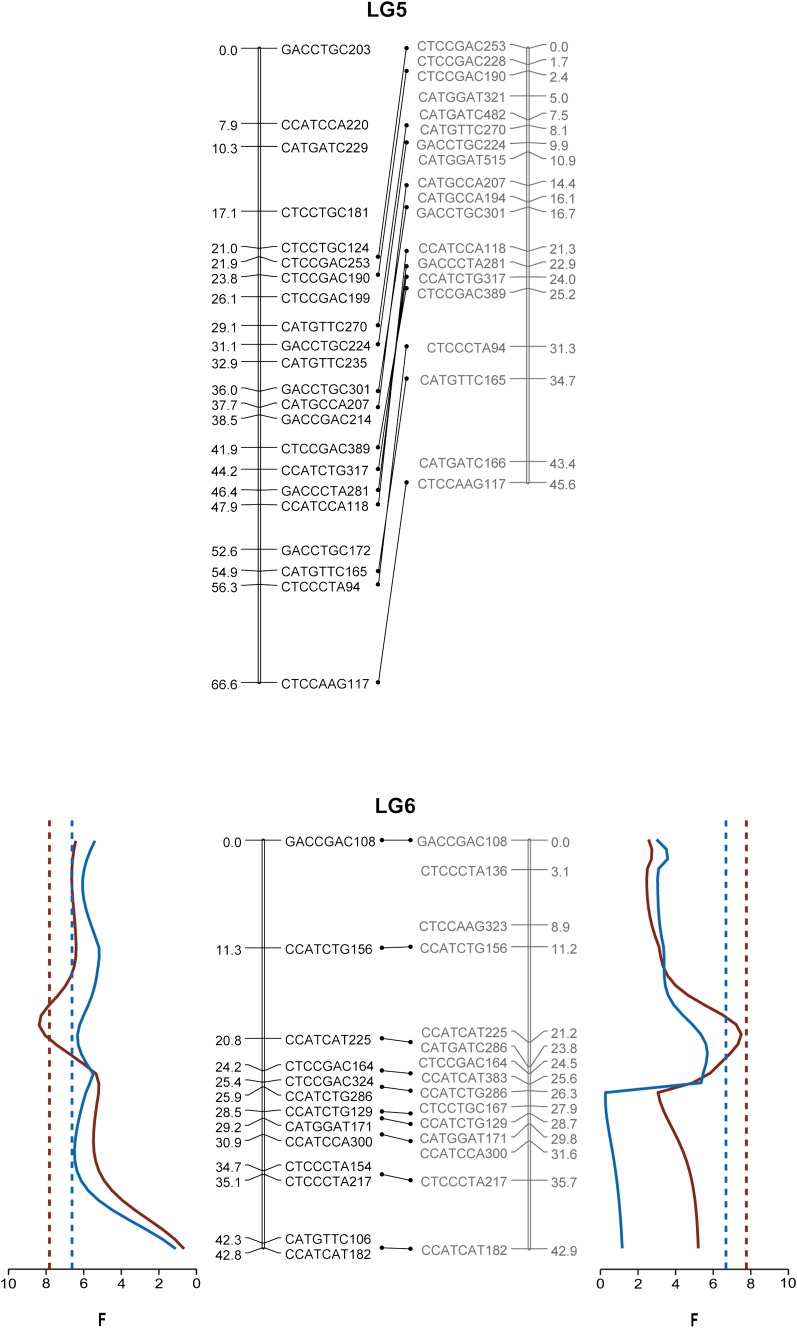
Estimated linkage map showing the positions of the detected QTL. Marker names and the corresponding relative genetic distances (cM) are shown alongside the vertical lines representing LGs. Pairs of linkage groups from the maternal linkage map (black) and the paternal linkage map (gray) are connected by markers segregating in both parents (note that LG2 from the maternal map does not have a corresponding linkage group in the paternal map). The profiles of the *F*-statistic are shown only for those LG showing suggestive or significant QTL (blue = body size; red = beak length). Dotted lines represent the 1% chromosome-wide significance thresholds as determined by permutation tests (see text).

Cytogenetically, the soapberry bug (*J. haematoloma*) is characterized by an XX/X0 (female/male) sex determination system, five pairs of autosomal chromosomes, and one pair of m chromosomes (2n_female_ = 10 + 2m + XX, 2n_male_ = 10 + 2m + X0; Bressa *et al.* 2001). The m chromosomes are small, achiasmatic, and behave as univalents during early meiotic stages (Bressa *et al.* 2001, 2005) and, *a priori*, we did not anticipate covering it in our linkage map. Accordingly, the Grouping function of JoinMap 4.0 split the maternal markers into six linkage groups and the paternal markers into five linkage groups at LOD of 4, encompassing 341 cM and 232 cM, respectively (Figure 3). Although the paternal map likely represents the five pairs of autosomes, the maternal map has an extra linkage group (LG2) that contains sex-determining QTL (data not shown), and we believe that it represents the X chromosome.

The recovered linkage groups in the maternal map ranged from 43 cM to 75 cM (mean, 56.8 cM) with an average density of 20.5 markers per group (range, 11–31). Similarly, the size of the linkage groups in the paternal map varied from 41 to 55 cM (mean, 45.3 cM) with a mean density of 17.2 markers per linkage group (range, 14–22). The mean distances between adjacent loci were similar in the maternal (mean ± SE, 2.91 cM ± 0.27 cM) and paternal (2.79 cM ± 0.31 cM) maps, and the longest distance between adjacent loci was 19.34 cM on the maternal LG 4. Thus, although in many species the frequency of recombination differs between sexes, we found no evidence that this is the case in *J. haematoloma*.

The total map length of *J. haematoloma* seems to be short for an insect with an estimated genome size of 1.79 Gb (J. A. Andrés, unpublished data). Several molecular and cytogenetic characteristics are likely to contribute to the observed reduced recombination rates: first, *J. haematoloma* autosomal bivalents usually show only one terminal chiasma (Bressa *et al.* 2001); second, chromosomes are holocentric, lacking centromeres, and this structure may be prone to reduced recombination (Bressa *et al.* 2001, 2005); third, at least in the male germ line univalent autosomes can be relatively common (Bressa *et al.* 2001). Our short linkage maps are consistent with these characteristics.

The behavior of the largest pair of autosomal chromosomes in *J. haematoloma* is noteworthy. This pair of chromosomes can be often observed as univalents or as a bivalent with terminal chiasmata, resulting in a large area of reduced recombination around the center of the chromosome (Bressa *et al.* 2001). Thus, one might *a priori* expect a map with at least one linkage group showing spatially aggregated markers around the center. The observed patterns are consistent with this prediction. Although in five (out of six) linkage groups the positions of the AFLP markers do not deviate significantly from a random distribution (χ^2^ goodness-of-fit, *P* > 0.05), LG 4 shows a significant aggregation of markers (*P* < 0.001) around the center in both the paternal and the maternal maps.

### Genetic architecture of beak and body length

When the genetic architecture of a trait is characterized by one or a few loci of large effect, rapid adaptation may be facilitated by the fixation of a few mutations in those loci (Orr and Coyne 1992; Orr 2005; Barrett and Schluter 2008). Thus, traits involved in contemporary evolution may be controlled by only a few genes with major effects on the phenotype. Such oligogenic models predict that beak length should be controlled by only a few QTL. Our interval mapping analysis using one-QTL model revealed only one significant QTL for beak length on LG 6 (maternally at *P*_experiment-wide_ < 0.01 and paternally at *P*_Chromosome-wide_ < 0.05). In both maps, the markers with the highest *F*-value associated with this QTL occupied the same position (maternal position: 19 cM; paternal position: 20 cM; Figure 3), and in both cases the detected QTL had a moderate effect (about 15%) on beak length (*V*_explained-maternal_ = 15.7%; *V*_explained-paternal_ = 14.1%). Using this QTL as background genetic effects, no more QTL could be found in any of the other linkage groups. Similarly, a two-QTL model also failed to find any other loci associated with beak length on LG 6. Including QTL × sex interactions in the one-QTL model had no significant effect on the residual phenotypic variance, suggesting that the effects of this QTL are similar in both sexes. Our single-marker regression analyses found eight markers associated with beak length. After controlling for sex differences, we found that the percentages of beak length variance explained by these markers ranged from 5 to 10% (average 7%, Table 1). Our results, therefore, are consistent with an oligogenic model in which the rapid evolution of beak length to better match the fruit size of a newly introduced host is controlled by a limited number of loci of substantial effect.

**Table 1:** Results of single-marker analysis using analysis of covariance on markers that were unlinked at LOD <4 (in bold) and markers with significant segregation distortion

Marker	Beak Length			Body Size		
	Allele Effect	*P* Value	%*^a^*	Allele Effect	*P* Value	%*^a^*
CCATCAT186	−0.08	0.021	6.7	0.11	0.009*^b^*	8.4
**CCATCTG199**	−0.09	0.036	5.5	−0.32	0.001	13.0
**CCATCTG241**	−*0.34*	0.020	6.4	ns	ns	ns
CTCCCTA146	−*0.34*	0.005	9.7	ns	ns	ns
CTCCCTA204	−*0.18*	0.022	6.6	ns	ns	ns
CTCCAAG178	−*0.11*	0.048	4.9	ns	ns	ns
CCATCGC293	−0.03	0.017	7.1	ns	ns	ns
GACCGAC234	0.18	0.008*^b^*	8.7	ns	ns	ns
**CATGTTC148**	ns	ns	ns	−*0.28*	0.035	5.6

Allele effect: a positive value indicates that the “dominant” allele (peak present) increases the trait value, and a negative value indicates that the dominant allele decreases the trait value; allele effect in italics: indistinguishable additive and dominance effects. Note that allele origins are unknown due to using of outbred lines. LOD, logarithm of odds; ns, nonsignificant.

a Percentage of the trait variation explained by this marker.

b A significant interaction between sex and marker was present.

However, the number and effect of QTL observed in our study have to be interpreted cautiously. First, the relatively small size of our mapping family results in a limited power to detect QTL of small effect and in an overestimate of the effect of the detected ones (Beavis 1998; Xu 2003). This is also true for QTL found in regions showing low recombination rates (Noor *et al.* 2001). Second, the number, position, and effect of QTL may be specific for the parental populations analyzed and further QTL may be found in different genetic backgrounds. Therefore, the located QTL could reflect simple within-population variation rather than evolved differentiation between ecomorphs (Bradshaw *et al.* 2012). These potential caveats do not necessarily compromise our results. However, further studies involving several crosses (and possibly recombinant inbred line populations) are needed to get a definitive picture of the number of genetic elements determining beak length in soapberry bugs.

From an adaptive perspective, differences in beak length are the most interesting because of the clear ecological relevance of this trait. Yet, bugs colonizing the introduced tree differ from the ancestral bugs in a variety of morphological and physiological traits. Previous studies have shown that although there are no significant differences in body size between bugs feeding on the introduced and native host (Carroll and Boyd 1992), hybrid lines with relatively longer beaks tend to be bigger (Carroll *et al.* 2001), suggesting that these two traits are not fully independent. The beak length QTL found on LG 6 colocalizes with a suggestive QTL for body size (*P*_Chromosome-wide_ = 0.02, *V*_explained_ = 12.2%; Figure 3), indicating either linkage disequilibrium between two different beak and body length QTL or a single QTL with pleiotropic effects. In this case, including QTL × sex interactions in the one-QTL model had significant effect on the residual body length variance, indicating that the effects of this QTL are different between sexes. Controlling for the effect of this QTL on LG 6, we found two more QTL related to overall body size differences in the maternal map (Figure 3). The first of these QTL is located on the putative X chromosome (LG 2) and had a moderate effect on body length (*P*_experiment-wide_ < 0.01, *V*_explained_ = 11.8%). The second one is located in LG1 (*P*_chromosome-wide_ < 0.01, *V*_explained_ = 5.9%). Single-marker regression analyses found three markers significantly associated with body length. After controlling for sex, we found that the percentages of body size variance explained by these markers ranged from 8 to 13% (average 9%). Interestingly, two of them (CCATCAT186 and CCATCTG199, Table 1) had significant effects on both beak length and body size. Even more interestingly, CCATCTG199 showed opposite effects on the two traits. This finding again shows that though developmentally and/or genetically interrelated, these two traits have different genetic architectures.

Our findings altogether revealed a complex genetic architecture underlying beak diversification in soapberry bugs. Former studies showed that differences in beak length involved a substantial amount of both additive and nonadditive, particularly epistatic, genetic variation (Carroll *et al.* 2001; Carroll 2007). Thus a *priori*, we expected to detect significant QTL × QTL interactions. In contrast, with the two-dimensional two-QTL genome scan using the Haley-Knott regression method in R/qtl, we could not find any potential QTL interactions for beak length. This apparent contradiction between our results and those of previous studies is likely to be the result of our low power to detect epistasis. Detecting epistasis is far more difficult than detecting single QTL and requires relatively big samples sizes (n > 400), especially in the case of interactions involving dominance effects (Mao and Da 2005; Wei *et al.* 2010). Dominance is an important component of variance in beak length in soapberry bugs (Carroll *et al.* 2001; Carroll 2007). Therefore, it is not entirely surprising that we could not detect any significant epistatic effects. Similarly, our two-dimensional two-QTL genome scan for body size could not detect any significant QTL interactions.

To conclude, contrasting views still exist on the number of underlying loci and magnitude of allelic effects involved in adaptation in natural populations. At the two ends of a continuum, adaptive evolution can be driven by changes in many genes of minor effect (polygenic model), or by mutations in a few genes of major effect (oligogenic model; Orr and Coyne 1992; Orr 2005). Although rapid large phenotypic shifts observed in Jadera beaks suggest the existence of loci of relatively large effects (Orr 2005; Barrett and Schluter 2008), comparative and experimental evidence (Carroll *et al.* 2003; Stern and Orgogozo 2009) also point toward the presence of small effect and epistatic loci. A major contribution of our work is the assessment of the number of loci involved in beak reduction. Our results suggest an oligogenic control of beak length.

Finally, our findings provide a framework for future identification of the genes responsible for rapid beak length differentiation using a combination of fine-mapping, a candidate gene approach, and functional analysis. Ultimately, these experiments will help us to understand the evolution of beak length differences associated with other anthropogenic host-shifts, such as the Australian red-eyed bug, *Leptocoris tagalicus*, which has colonized two introduced species of invasive balloon vines that have much larger fruits than the native hosts (Carroll 2007).
